# High Dose Vitamin D supplementation alters faecal microbiome and predisposes mice to more severe colitis

**DOI:** 10.1038/s41598-018-29759-y

**Published:** 2018-07-31

**Authors:** Simon Ghaly, Nadeem O. Kaakoush, Frances Lloyd, Terence McGonigle, Danny Mok, Angela Baird, Borut Klopcic, Lavinia Gordon, Shelley Gorman, Cynthia Forest, Roger Bouillon, Ian C. Lawrance, Prue H. Hart

**Affiliations:** 1 Telethon Kids Institute, The University of Western Australia, Perth, WA Australia; 2 School of Medicine and Pharmacology, The University of Western Australia, Perth, WA Australia; 3 Department of Gastroenterology and Hepatology, St. Vincent’s Hospital, Sydney, NSW Australia; 4 School of Medical Sciences, UNSW Sydney, Kensington, NSW Australia; 5 Australian Genome Research Facility, The Walter and Eliza Hall Institute, Parkville, Victoria Australia; 6 Department of Anatomical Pathology, PathWest, Fiona Stanley Hospital, Murdoch, WA Australia; 7 Clinical and Experimental Endocrinology, Katholieke Universiteit Leuven, Leuven, Belgium; 8 Centre for Inflammatory Bowel Disease, St. John of God Hospital, Subiaco, WA Australia

## Abstract

Vitamin D has been suggested as a possible adjunctive treatment to ameliorate disease severity in human inflammatory bowel disease. In this study, the effects of diets containing high (D++, 10,000 IU/kg), moderate (D+, 2,280 IU/kg) or no vitamin D (D−) on the severity of dextran sodium sulphate (DSS) colitis in female C57Bl/6 mice were investigated. The group on high dose vitamin D (D++) developed the most severe colitis as measured by blinded endoscopic (p < 0.001) and histologic (p < 0.05) assessment, weight loss (p < 0.001), drop in serum albumin (p = 0.05) and increased expression of colonic TNF-α (p < 0.05). Microbiota analysis of faecal DNA showed that the microbial composition of D++ control mice was more similar to that of DSS mice. Serum 25(OH)D_3_ levels reduced by 63% in the D++ group and 23% in the D+ group after 6 days of DSS treatment. Thus, high dose vitamin D supplementation is associated with a shift to a more inflammatory faecal microbiome and increased susceptibility to colitis, with a fall in circulating vitamin D occurring as a secondary event in response to the inflammatory process.

## Introduction

Vitamin D is recognized as a regulator of both innate and adaptive immune responses^1^, and vitamin D deficiency has been associated with the development of a number of immune mediated disorders including the inflammatory bowel diseases (IBD), Crohn’s disease (CD) and ulcerative colitis (UC).

Several confounding factors, such as reduced sunlight exposure, low dietary intake and reduced intestinal absorption, limit the ability to draw conclusions about the causality of the observed link between vitamin D deficiency and active IBD. As a result, investigators have turned to mouse models of IBD such as the dextran sodium sulphate (DSS)-induced model of colitis^2^. Vitamin D receptor (VDR) knockout (KO), CYP27B1 KO, as well as dietary vitamin D-deficient mouse models are more susceptible to colitis^3–5^. In an IL-10-KO mouse model, which typically develop spontaneous colitis, administration of 1,25(OH)_2_D ameliorated the severity of colitis^6^. Thus, in select animal models, vitamin D deficiency increases susceptibility to colitis, and restoring vitamin D sufficiency may ameliorate colitis.

There are few studies exploring the effect of high vitamin D levels on immune regulation. Population studies describe a reverse ‘J’ or ‘U’ phenomenon where both vitamin D deficiency and high vitamin D are associated with increased all-cause and cardiovascular specific mortality^7–9^. Higher vitamin D levels at birth have also been linked to the development of allergy^10,11^, and genetic analysis within these cohorts identify epigenetic changes in a number of genes including the thymic stromal lymphopoietin (*TSLP*) gene that may explain the immune mechanism for predisposition to allergy with elevated vitamin D levels^12^. The effect of higher vitamin D levels in clinical IBD or animal models of IBD have not been explored.

Vitamin D-related changes to gut microbiota are a possible mechanism for altering susceptibility to colitis. Vitamin D-deficient mice developed elevated bacterial counts in colonic tissue and greater susceptibility to DSS colitis^3^. Dietary-induced vitamin D deficiency alters the composition of the faecal microbiome of C57Bl/6 mice, with an increase in the relative quantities of Bacteroidetes, Firmicutes, Actinobacteria, and Gamma-Proteobacteria in naïve, non-colitic mice^13^. The effect of vitamin D on microbiota, however, is not limited to the gastrointestinal tract with an inverse correlation between circulating 25(OH)D levels and *Pseudomonas* operating taxonomic units (OTU) observed in the lungs of naïve female BALB/c mice^14^. Thus, vitamin D may regulate the microbiome at different sites and this could be due to its effect on innate immune responses, in particular the expression of antimicrobial peptides, such as the cathelicidins and β-defensins^15^.

The aim of this study was, therefore, to determine the effect of diets supplemented with high doses of vitamin D, compared to standard diets sufficient in vitamin D and diets deficient in vitamin D with no supplementation, on the susceptibility to DSS-induced colitis. Further, we sought to investigate the effect of different doses of vitamin D on the faecal microbiota and how this correlated with susceptibility to colitis.

## Materials and Methods

### Mice and Diet

Female 6 week-old C57Bl/6 mice were fed semi-pure diets supplemented with higher than usual doses of vitamin D (SF14-069, Specialty Feeds, Perth, Western Australia, 10,000 IU/kg vitamin D_3_, 0.5% calcium), standard doses of vitamin D to achieve vitamin D sufficiency similar to standard chow (SF05-34, Specialty Feeds, 2,280 IU/kg vitamin D_3_, 1% calcium) or no added vitamin D to induce vitamin D deficiency (SF05-033, Specialty Feeds, 0 IU/kg vitamin D_3_, 2% calcium). All experiments were performed according to the ethical guidelines of the National Health and Medical Research Council of Australia with the approval from the Telethon Kids Institute Animal Ethics Committee (AEC #276). Mice were purchased from the Animal Resources Centre, Western Australia.

Mice were housed under perspex-filtered fluorescent lighting, which emitted no detectable UVB radiation as measured using a UV radiometer (UVX Digital Radiometer, Ultraviolet Products Inc., Upland, CA, USA).

### Colitis Model

**Figure 1:**
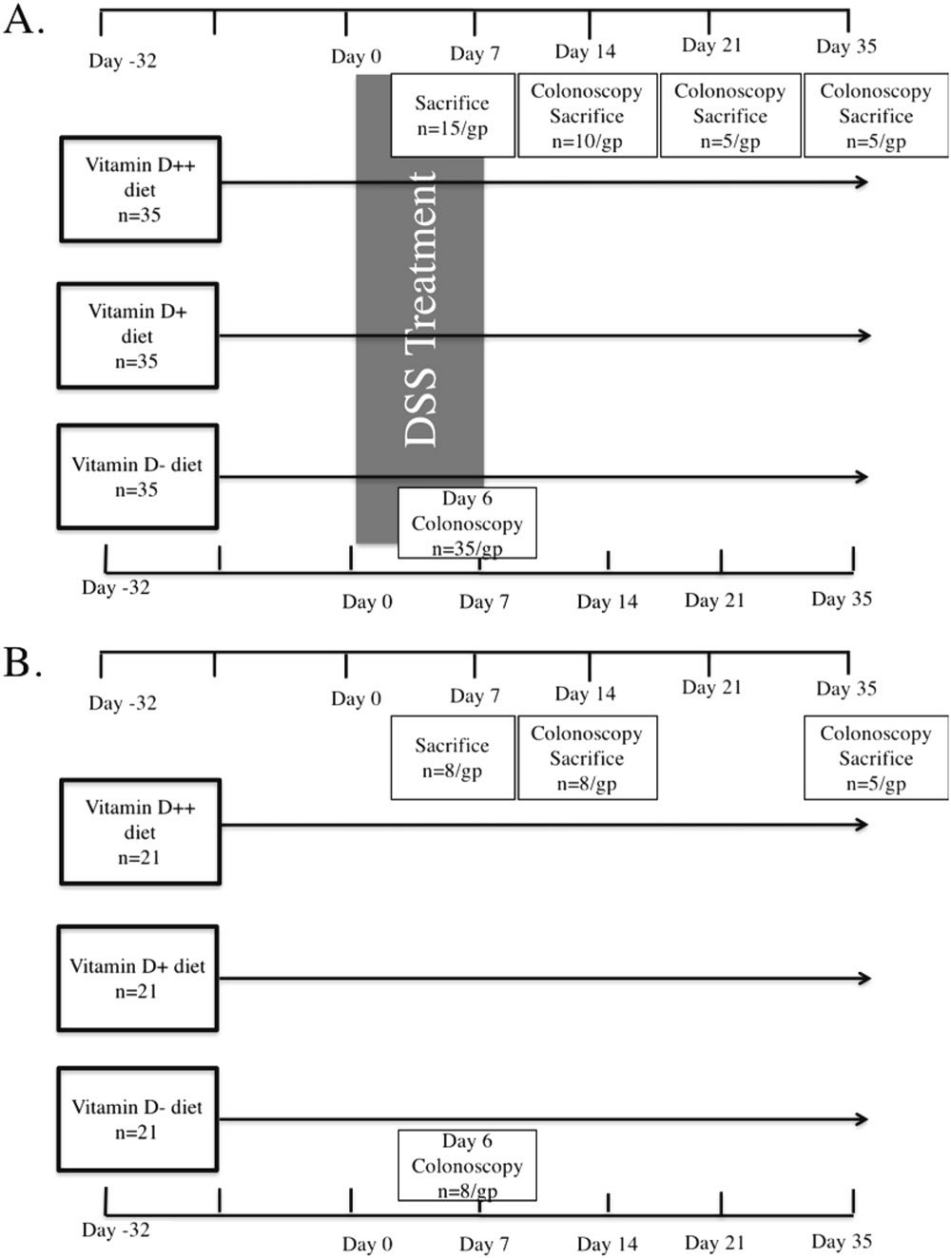
The experimental approach. 6-week-old C57BL/6 female mice were fed diets with high (D++), moderate (D+) or no (D−) vitamin D3. (**A**) After 4 weeks of being fed the respective diets, mice were treated with DSS for 6 days to induce colitis, and then were maintained without DSS and continued their respective vitamin D diet until day 35. All mice underwent colonoscopy at day 6, and were sacrificed at day 7, 14, 21 or 35. An additional colonoscopy was performed on day 14, 21 or 35 when mice were sacrificed. (**B**) A control group of mice fed the three different diets followed the same protocol but were not treated with DSS and the day 21 timepoint is not included. The experiment was performed twice.

After 4 weeks of being fed the respective diets, colitis was induced by the addition of DSS [3% (wt/vol)(MP Biomedicals LLC, OH)] to the drinking water for 6 days. Control mice received water without DSS. As the efficacy of DSS varies between batches, the experiments were conducted using the same batch^16^. In preliminary experiments, 3% DSS induced adequate colitis with peak weight loss ranging between 0.3% to 10.3% at day 7. Following induction of colitis, mice recovered over a period of 0–4 weeks without ongoing DSS treatment (Fig. 1). Mouse body weight was assessed daily during DSS treatment and weekly during recovery. The experiment was repeated, with a total of 35 mice per group. Mice were sacrificed at day 7, 14, 21 and 35.

### Murine colonoscopy

A high-resolution mouse video endoscopic system was used to assess the level of colitis. All mice were scoped on day 6 after commencing DSS treatment and then at the time of sacrifice. Mice were anaesthetised using isofluorane unless the colonoscopy was being performed at the end-point when ketamine 20 mg/ml and xylazine 2 mg/ml by intraperitoneal injection was used. All procedures were digitally recorded then scored in a blinded fashion. The experimental endoscopy setup consisted of a miniature endoscope (1.9 mm outer diameter), a xenon light source, a triple chip camera, and an air pump (Karl Storz, Germany) to achieve regulated inflation of the mouse colon.

The severity of colitis was determined using the modified ‘**m**urine **e**ndoscopic **i**ndex of **c**olitis **s**everity’ (MEICS)^16,17^. The MEICS system consists of 5 parameters: thickening of the colon wall, changes of the normal vascular pattern, presence of fibrin, mucosal granularity and stool consistency. Endoscopic grading was performed for each parameter (scored between 0 and 3) leading to a cumulative score of between 0 (no signs of inflammation) and 15 (endoscopic signs of severe inflammation). Healthy mice had a score of 0–3.

### UV Radiation

For UV experiments, a bank of six 40 W lamps (Philips TL UV-B, Eindhoven, The Netherlands) emitting broadband UVR, 250–360 nm, with 65% of the output in the UVB range (280–315 nm), was used to irradiate mice and to deliver 1 kJ/m^2^ of UVR onto clean-shaven 8 cm^2^ dorsal skin. A new sheet of PVC plastic film (0.22 mm) was taped to the top of each Perspex cage immediately before irradiation to screen wavelengths <290 nm. Sunlamps were held 20 cm above the cages.

### Histological Assessment of Colitis

Colons were removed with the rectum discarded as this has a different tissue fibro-structure. The distal 1 cm of colon was dissected, cleaned, formalin-fixed and embedded in paraffin wax. Sections were stained with haemotoxylin and eosin (H&E). All H&E sections were assessed blindly by a specialist gastroenterological histopathologist (CF) according to the scoring system by Dieleman *et al*.^18^. In this scoring system, the severity and depth of inflammation as well as the level of crypt damage and regeneration are scored.

### Measurement of serum metabolites

At the time of sacrifice, blood was drawn by cardiac puncture. Levels of 25(OH)D_3_ were measured in the serum by liquid chromatography tandem mass spectroscopy (LC/MS/MS) (Centre for Metabolomics, UWA)^19^. Levels of 1,25(OH)_2_D_3_ were measured using IDS EIA ELISA kits (Immunodiagnostic Systems Ltd, Fountain Hills, AZ) as described by the manufacturer.

The serum vitamin D binding protein (VDBP) concentration was measured in duplicate using a Quantikine ELISA (R&D Systems, Minneapolis, MN) as per the manufacturer’s instructions. The results were verified by a radial immunodiffusion method as previously published^20^.

Serum calcium and albumin were measured by standard colorimetric reactions using the Architect c16000 Analyzer (Abbott Diagnositcs, Abbott Park, IL) by PathWest, Royal Perth Hospital, WA.

Serum cytokines were measured using Bio-Plex Pro™ Mouse Cytokine 23-plex panel (Bio-Rad Laboratories, Hercules, CA) as per the manufacturer’s instructions. The cytokines analysed included interleukin (IL)-1α, IL-1β, IL-2, IL-3, IL-4, IL-5, IL-6, IL-9, IL-10, IL-12 p40, IL-12 p70, IL-13, IL-17, eotaxin (CCL11), G-CSF, GM-CSF, IFN-γ, KC, MCP-1, MIP-1α, MIP-1β, RANTES and TNF-α.

### Real-time PCR

Messenger RNA was extracted from snap-frozen colon, liver and kidney tissue with cDNA synthesised and real-time assays performed as previously described^21^. Real-time PCR primers were *CYP27B1* cat # 301447280210/0&1, *CYP24A1* cat # 3014472802-20/0&1, *CYP2R1* cat # QT0005750 (Sigma-Aldrich co., St. Louis, MO), and for TNFα Qiagen Quantitect Primer Assay (Qiagen, Hilden, Germany). Housekeeping genes included elongation factor 1α (eEF1α) for kidney and liver tissue, TATA-box-binding protein for colonic tissue (Sigma-Aldrich co., St. Louis,MO). Quantitect SYBRGreen was used for qPCR on the AB17900HT instrument. Fold-change was determined by using the 2^−∆∆Ct^ method.

### Faecal Microbiota analysis

The faecal microbiome was analysed by sequencing the V3-V4 segment of the 16S ribosomal RNA (rRNA) gene using Illumina MiSeq chemistry. Faecal pellets were collected and stored at −20 °C. Bacterial DNA was extracted using the PowerSoil® DNA isolation kit according to the manufacturer’s instructions (MO BIO Laboratories, Carlsbad, CA). PCR amplification (341F/806F primer pair) and sequencing was performed by the Australian Genome Research Facility on the Illumina MiSeq (San Diego, CA) with 2 × 300 bp paired-end chemistry. Paired-ends reads were assembled by aligning the forward and reverse reads using PEAR (version 0.9.5)^22^. Primers were trimmed using Seqtk (version 1.0)^23^. Trimmed sequences were processed using Quantitative Insights into Microbial Ecology (QIIME 1.8)^24^ USEARCH (version 7.1.1090)^25,26^ and UPARSE^26^ software. Using QIIME, taxonomy was assigned using Greengenes database (Version 13_8, Aug 2013)^27^.

The biom file, the OTU table, the taxonomic assignments and associated sample data were imported into R to create a phyloseq object. For all beta diversity analyses, OTUs for which the variance across all samples were very low, were filtered out. For testing a single categorical experimental condition, exact tests for differences in the means between two groups of negative-binomially distributed counts were computed. Data were normalized using the RLE scaling factor method and dispersions estimated. The counts were extracted and ranked by p-value, applying a false discovery rate cut-off of less than 0.001.

### Statistical Analysis

Statistical significance was calculated using IBM® SPSS® Statistics Version 22 (IBM Corp. Armonk, NY). All graphs and comparison of differences between groups were assessed using Student’s unpaired t-test or ANOVA with post hoc LSD analysis for multiple group analysis. Non-parametric data using Mann-Whitney U and Kruskall-Wallis testing. Microbiome statistical analysis was undertaken using the programming language R, specifically the *phyloseq* and *edgeR* packages available through Bioconductor, a project providing tools for the analysis and comprehension of high-throughput genomic DNA. LEfSe (Linear discriminant analysis effect size) was used to identify differentially abundant microbial taxa^28^.

## Results

### Vitamin D_3_ diets

Six-week-old C57Bl/6 female mice were fed diets with high (D++), moderate (D+) or no (D−) vitamin D_3_ for four weeks. At ten weeks of age, the mice in the three groups were not significantly different in weight (data not shown). D++ mice had significantly higher serum 25(OH)D_3_ compared to D+ mice which was in turn higher than D− (Table 1). The serum concentration of activated vitamin D, 1,25(OH)_2_D_3_, was not significantly different between the D++ and D+ groups. As previously observed^29^, 1,25(OH)_2_D concentrations in the D− mice were lower than in the other groups, though this did not reach statistical significance (p = 0.087). There was no difference in serum calcium levels between the three groups.

**Table 1:** Serum 25 (OH)D, 1,25(OH)_2_D and calcium levels after 5 weeks on vitamin D diets.

	D++	D+	D−	p-value D++ vs D+	p-value D+ vs D−
25(OH)D nmol/L	100.8 ± 4.6	41.2 ± 2.0	12.0 ± 4.0	<0.001	0.001
1,25(OH)_2_D pmol/L	119.9 ± 17.7	140.3 ± 26.3	85.6 ± 8.6	NS	NS
Calcium mmol/L	2.2 ± 0.04	2.3 ± 0.1	1.9 ± 0.1	NS	NS

Data are shown as mean ± SEM for *n* = 7–8 for 25(OH)D levels, *n* = 3–5/group for 1,25(OH)_2_D, *n* = 4–5/group for calcium. *P values* reflect differences in mean between vitamin D groups calculated by independent student t-test. Results pooled from two independent experiments. NS = not significant.

### Vitamin D and DSS-induced colitis

**Figure 2:**
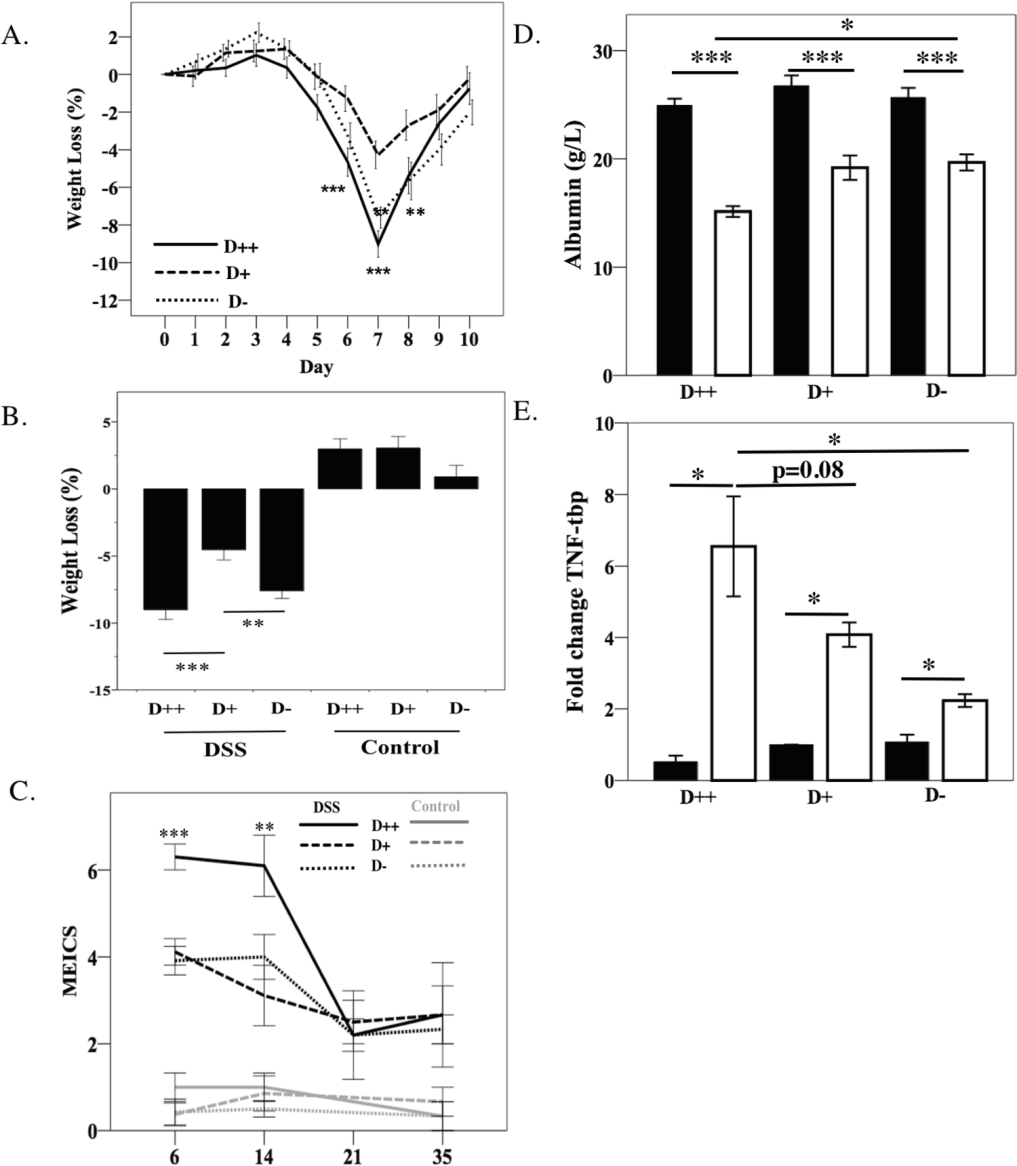
Outcomes of colitis. 6-week old female C57Bl/6 mice were established on diets with high (D++), moderate (D+) or no (D−) vitamin D3, before being treated with DSS for 6 days. Mice were regularly weighed and underwent colonoscopy procedures at regular intervals. (**A**) The percentage weight loss from baseline to day 10 post-DSS treatment. Comparisons are made to group D+ as the reference group. (**B**) Weight loss at day 7. (**C**) Endoscopic severity over time measured by murine endoscopic index of severity (MEICS). Comparisons are made to group D+ as the reference group. n = 35/group for day 6 and 7 assessments, n = 10/group day 14, n = 5/group for days 21 and 35. (**D**) Serum albumin at day 7, n = 5–10/group. (**E**) Colonic TNF-α gene expression after 6 days DSS treatment, fold change using the 2^−ΔΔT^ method with TATA-box-binding protein as housekeeping gene, n = 4–5/group. Solid bars for control mice, open bars for DSS mice. Data are shown as mean ± SEM, from two experiments. ^*^P < *0.05*, ^**^P < *0.01*, ^***^P < *0.001*.

Body weight loss and liquid stools were observed between 2–7 days after commencing the DSS treatment. All DSS-treated mice lost weight compared to pre-DSS measures. The peak percentage weight loss occurred at day 7 (Fig. 2A). At day 6 and 7, the D++ mice lost significantly more weight than D+ mice (p < 0.001), and at day 7 and 8, D− mice lost significantly more weight than D+ mice (p < 0.01) (Fig. 2A,B).

At the day 6 colonoscopy, control mice in all groups had solid stool, preserved mucosal vascularity, normal colonic translucency with a mean MEICS of 0.6 ± 0.17 (n = 23, range 0–3), consistent with no colitis (Fig. 2C). DSS-treated mice demonstrated endoscopic inflammation with loose stools, loss of intestinal wall translucency and mucosal bleeding with a mean MEICS 4.8 ± 0.2 (n = 102, range 0–10). This was significantly greater than control mice (p < 0.001, 95% CI 3.6–4.7).

At day 6, MEICS was significantly greater in D++ (6.3 ± 0.30, n = 33) than in the D+ (4.1 ± 0.30, n = 34, p < 0.001) and D− (3.9 ± 0.33, n = 35, p < 0.001) groups (Fig. 2C). There was no difference in colitis severity observed in mice from the D+ and D− groups (p = 0.65). At day 14, a higher MEICS was observed in the D++ group compared to the D+ group (p < 0.01), but this difference resolved by day 21 and 35 as recovery was almost complete (Fig. 2C).

The histological grading of colitis at the distal colon on day 14 was greater in mice from the D++ (8.22 ± 2.53, n = 9) than D+ (1.42 ± 0.57, n = 7, p < 0.05) or D− (1.43 ± 0.57, n = 8, p < 0.05) groups. At Day 7 there was a trend for higher inflammation in D++ and D− compared to D+ mice though this did not reach statistical significance (p = 0.25) (Supplementary Fig. 1A,B). Among all groups, there was positive correlation between MEICS and day 7 weight loss (r = 0.60, n = 253, p < 0.001), day 7 histological score (r = 0.51, n = 86, p < 0.001) and day 14 histological score (r = 0.59, n = 56, p < 0.001) (data not shown).

At day 7, the mean serum albumin was less in all groups with colitis (Fig. 2D) than corresponding controls. Among DSS-treated mice, the mean albumin level was significantly lower among the D++, compared to the D+ (p < 0.05) and D− (p < 0.05) group, consistent with a worse colitis seen in the D++ group.

Gene expression of TNF-α in colon tissue by RT-PCR at day 7, was increased in all DSS-treated groups compared to controls. This was again greatest in the D++ group compared to D− (6.5 ± 3.1 vs 2.2 ± 0.36 fold, p < 0.05) with a trend to be greater than that measured in the D+ mice (4.1 ± 0.11, p = 0.08) (Fig. 2D).

Serum cytokines TNF-α, IFN-γ, IL-10, IL-6, IL-12p40 and IL-1β at day 7 were greater among all DSS mice compared to control mice (p < 0.05 for all cytokines, data not shown). When comparisons are stratified by vitamin D groups, the DSS group had higher levels than controls though this was not always statistically significant (Supplementary Fig. 2). IL-12p40 was highest among D++ compared to D− groups (p < 0.01), but similar changes were not seen with other cytokines (Supplementary Fig. 2).

### 25(OH)D, 1,25(OH)_2_D and VDBP concentrations

**Figure 3:**
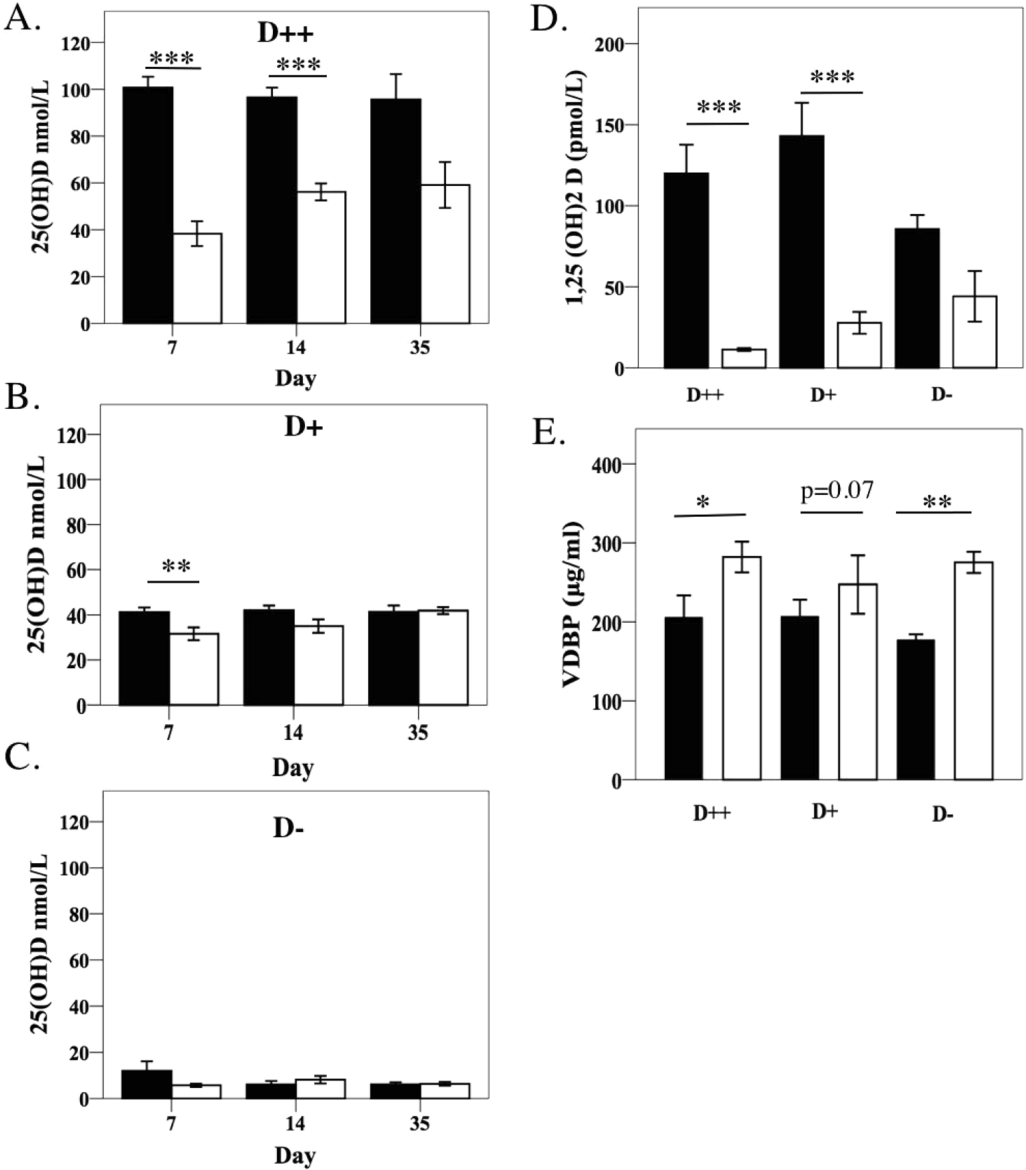
Vitamin D and vitamin D binding protein levels. Serum concentrations of 25(OH)D_3_ at day 7, 14 and 35 among DSS-treated mice and controls, (**A**) D++, (**B**) D+, (**C**) D−, *n* = 7–8/group at day 7 and day 14, *n* = 5/group at day 35. (**D**) 1,25(OH)_2_D and (**E**). Vitamin D binding protein concentrations in serum at day 7, *n* = 3–5/group. Solid bars for control mice, open bars for DSS mice. Values are expressed as mean ± SEM, from at least two experiments. ^*^P < *0.05*, ^**^P < *0.01*, ^***^P < *0.001*.

At day 7, serum 25(OH)D reduced by greater than 60% among DSS mice from the D++ group (Fig. 3A). This difference remained at day 35 (p = 0.053, n = 5). At day 7 a similar but smaller reduction in serum 25(OH)D levels was observed in the D+ group (Fig. 3B). Differences were not seen at the later time points. In mice from the D− group, given that 25(OH)D levels were already low at baseline, no reduction in 25(OH)D was detectable (Fig. 3C).

As the large decrease in 25(OH)D may have been due to an increase in its conversion to 1,25(OH)_2_D, changes in the levels of 1,25(OH)_2_D were investigated. A significant decrease at day 7 in 1,25(OH)_2_D concentrations (relative to control mice), was detected in both the D++ and D+ groups suggesting that increased conversion was not the cause of 25(OH)D reductions (p < 0.001) (Fig. 3D).

As 25(OH)D, and to a lesser extent 1,25(OH)_2_D, are mostly bound to the VDBP, we questioned if the large decrease in both these vitamin D metabolites was due to a loss of VDBP. As described previously (Fig. 2D), serum albumin dropped with colitis. Surprisingly, the VDBP levels, measured by ELISA, increased with colitis in all groups and this was statistically significant in the D++ and D− groups (Fig. 3E). The increase in VDBP with DSS colitis was also seen when VDBP was measured by radial immunodiffusion, though this was significant among the D+ and D− group and there was a trend to significance among the D++ group (p = 0.076) (Supplementary Fig. 1C). These data suggest that the induction of colitis increases circulating VDBP levels.

### Kidney *CYP24A1* gene expression in DSS colitis

**Figure 4:**
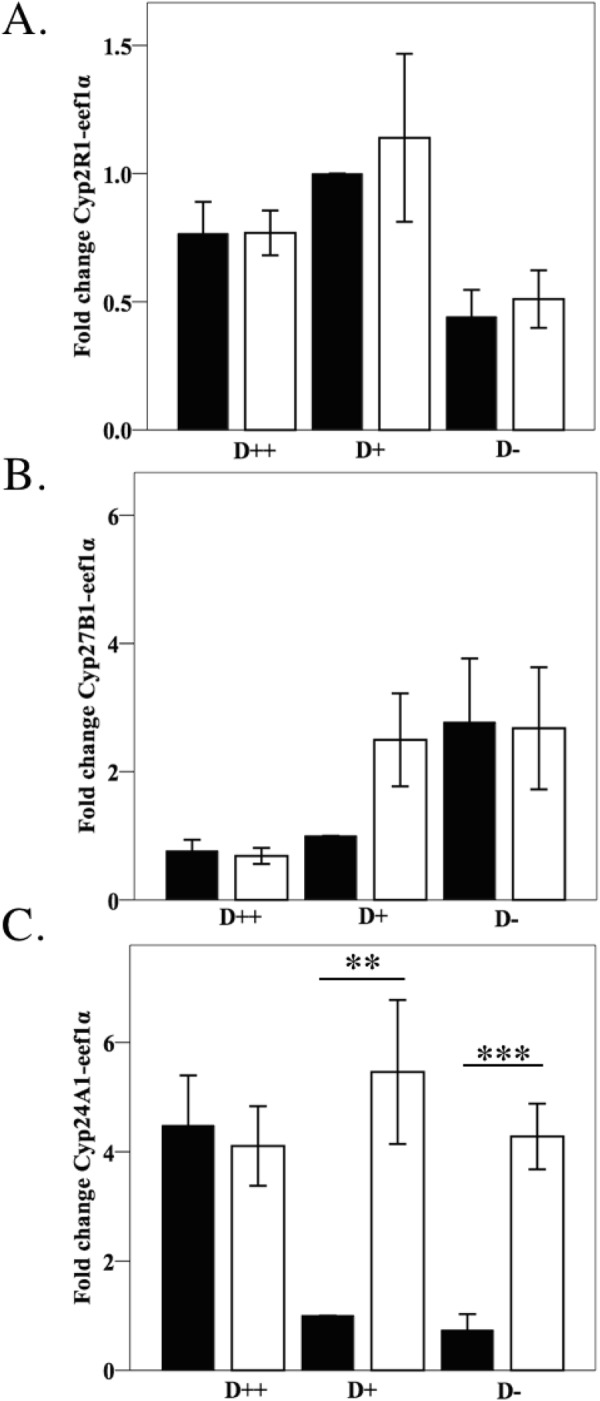
Kidney Cyp24A1 gene expression is upregulated with DSS colitis. Female C57Bl/6 mice were established on three vitamin D diets for 4 weeks before treatment with DSS. On day 7 mice were sacrificed with livers and kidneys harvested to determine (**A**) Liver Cyp2R1, (**B**) Kidney Cyp 27B1, and (**C**). Kidney Cyp24A1 gene expression. mRNA gene expression by qtPCR was calculated using the 2^−ΔΔCT^ method with eEF1α as the housekeeping gene, *n* = 5–10/group. Solid bars for control mice, open bars for DSS mice. Values are expressed as mean ± SEM, from at least two experiments. ^*^P < *0.05*, ^**^P < *0.01*, ^***^P < *0.001*.

In an attempt to explain the reduced 25(OH)D and 1,25(OH)_2_D at day 7 post-DSS treatment, changes in the level of expression of mRNA of enzymes involved in vitamin D metabolism were explored. Neither liver *CYP2R1* nor kidney *CYP27B1* mRNA levels changed significantly with the induction of colitis (Fig. 4A,B). There was 5.5 ± 1.3 fold more *CYP24A1* mRNA in the kidneys of DSS-treated mice on D+ diets compared to their corresponding control group (p < 0.01) (Fig. 4C). Similarly, kidney *CYP24A1* mRNA was expressed 4.3 ± 0.6 fold more among DSS-treated mice on D− diets compared to corresponding controls (p < 0.001). Kidney *CYP24A1* was up-regulated 4.5 ± 0.9 fold among D++ controls compared to D+ controls (p < 0.05) as an appropriate homeostatic mechanism, but there was no further increase with the induction of colitis. Thus, increased kidney metabolism may help to explain the reduced 25(OH)D and 1,25(OH)_2_D at day 7 in the D+ group, though a yet to be identified mechanism must exist to explain the drop in the D++ group.

### UV radiation-induced 25(OH)D and colitis

The reduced circulating levels of 25(OH)D and 1,25(OH)_2_D observed in mice with DSS-induced colitis could be caused by decreased intestinal absorption of vitamin D. If so, then 25(OH)D derived from skin exposed to UVB radiation should not fall with inflammation. To test this, mice fed vitamin D-deficient diets for 4 weeks were treated with daily UV radiation (1 kJ/m^2^) for 4 days followed by biweekly UV (1 kJ/m^2^) for the remainder of the experiment (D−UV+). After the 4 days of UV pretreatment, mice were treated with DSS for a further 6 days.

**Figure 5:**
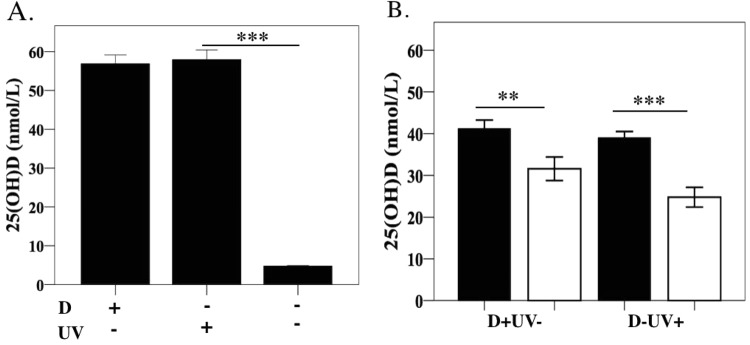
Serum levels of 25(OH)D_3_ in mice treated with and without UV radiation. The shaved dorsal surfaces of female C57Bl/6 mice on vitamin D deficient diets (D−) were irradiated with 1 kJ/m^2^ ultraviolet radiation daily for 4 days before undergoing DSS treatment. (**A**) 25(OH)D_3_ levels 24 h after four doses of UVB irradiation. (**B**) 25(OH)D levels after 6 days of DSS treatment in a separate group of mice. Solid bars control mice, open bars DSS mice. Values are expressed as mean ± SEM, from two experiments. *n* = 4/group day 0 and *n* = 7–8/group day 7. ^**^P < *0.01*, ^***^P < *0.001*.

After 4 doses of UV irradiation, serum levels of 25(OH)D in mice from the D−UV+ treatment was 58.0 ± 2.49 nmol/L compared to 4.8 ± 0.15 nmol/L among D− mice without UV treatment (D−UV−) (n = 4/group, p < 0.001), Fig. 5A. The 25(OH)D concentrations among D−UV+ mice were similar to the D+ without UV treatment (D+UV−).

After 6 days of DSS treatment, there was no significant difference in endoscopic severity of colitis between vitamin D-deficient mice (D−) exposed (UV+) or not exposed (UV−) to UV radiation (MEICS 4.3 ± 0.45 vs 3.9 ± 0.33, n = 35/gp, p = 0.42), nor was there a difference compared to D+UV− mice (MEICS 4.3 ± 0.45 vs 4.12 ± 0.45, n = 35/group, p = 0.64), Supplementary Fig. 1D. By day 7, the 25(OH)D (Fig. 5B) and 1,25(OH)_2_D concentrations (not shown) were significantly lower among UV-irradiated vitamin D-deficient, DSS-treated mice as compared to corresponding controls. Thus, these data suggest that the drop in circulating 25(OH)D in mice where vitamin D is acquired only through irradiation of the skin, cannot be due to malabsorption.

### Effect of vitamin D on faecal microbiota

Microbiota analysis was performed on 42 faecal samples, comprised of 5 samples from each of the vitamin D groups among controls and DSS mice at day 7 and 4 samples per group among controls at day 35. One control mouse was considered an outlier and excluded from further analyses (Supplementary Fig. 3A).

#### Effect of Vitamin D on faecal microbiota from control, non-DSS mice

**Figure 6:**
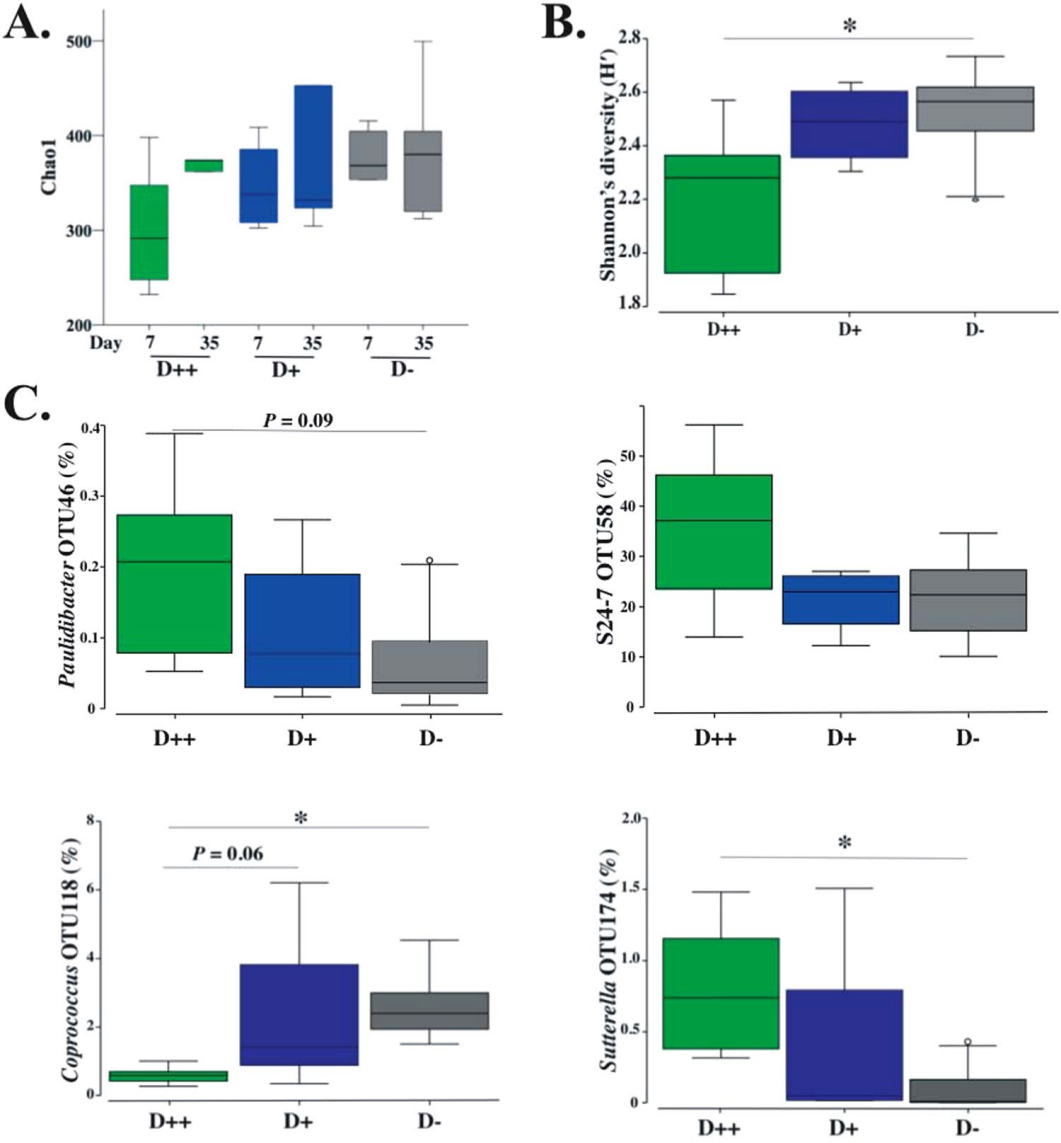
Microbial composition of faecal samples from control mice. Faecal pellets were collected control mice from each of the vitamin D dietary groups (D++, D+ and D−). (**A**) Comparison of day 7 and day 35 species richness for samples from control mice (measured by chao1). (**B**) Day 7 Shannon’s diversity (H′). (**C**) Day 7, Relative abundance (%) of OTUs that correlated with serum vitamin D levels among controls using distance based linear modeling (DistLM) analysis. P-values calculated by PERMANOVA on Euclidean distance resemblance matrices generated from square root transformed relative abundance from each OTUs. n = 5/group for day 7 analyses, n = 4/group at day 35. ^*^P < *0.05*.

There were no differences seen in α-diversity as measured by species richness, evenness or Shannon’s diversity in day 7 samples collected from plain water-treated control mice, and the result was reproducible for day 35 samples (Fig. 6A, Supplementary Fig. 3B,C). Similarly, no significant differences in β-diversity were noted between day 7 and day 35 samples from control mice (data not shown). Further analysis was carried out only on day 7 samples.

Comparisons between vitamin D groups found increasing vitamin D doses did not affect species richness as measured by chao1 among control mice (Fig. 6A), but it did reduce Shannon’s diversity between D++ controls compared to D− controls (Fig. 6B). No significant difference was noted between D− and D+ control groups; however, PERMANOVA analysis, a measure of global β-diversity, confirmed significant differences between D− and D++ controls (p = 0.012, t = 1.68, Permutations = 126) and D+ and D++ controls (P = 0.01, t = 1.76, Permutations = 126).

To examine the effect of vitamin D grouping on individual taxa, linear discriminant analysis (LEfSe) was performed. Forty microbial taxa at all taxonomic levels were found to be significantly different between the three vitamin D groups, of which 37 showed strong associations (linear discriminant analysis score > 3) (Supplementary Table Ia).

To determine the effect of measured serum 25(OH)D_3_ levels on individual taxa, correlation analysis was measured using distance based linear modelling (DistLM) analysis between serum 25(OH)D3 levels (Euclidean distance resemblance matrix) and relative abundances of microbial taxa. This identified a significant correlation with four taxa (>0.1% average relative abundance) which included: *Paulidibacter*|OTU46 (Pseudo-F: 4.6, P = 0.04, Df: 26); Bacteroidales|S24-7|OTU58 (Pseudo-F: 6.7, P = 0.02, Df: 26); *Sutterella*|OTU174 (Pseudo-F: 5.1, P = 0.038, Df: 26); and *Coprococcus*|OTU118 (Pseudo-F: 4.8, P = 0.02, Df: 26). To further inform our 25(OH)D3 correlation analyses and establish the response of these four taxa to vitamin D intake, the relative abundance of these four taxa across each vitamin D diet group were plotted (Fig. 6C). The relative abundance of *Paulidibacter*|OTU46, Bacteroidales|S24-7|OTU58, and *Sutterella*|OTU174 increased with vitamin D intake, while *Coprococcus*|OTU118 decreased.

#### Effect of DSS colitis on faecal microbiota

**Figure 7:**
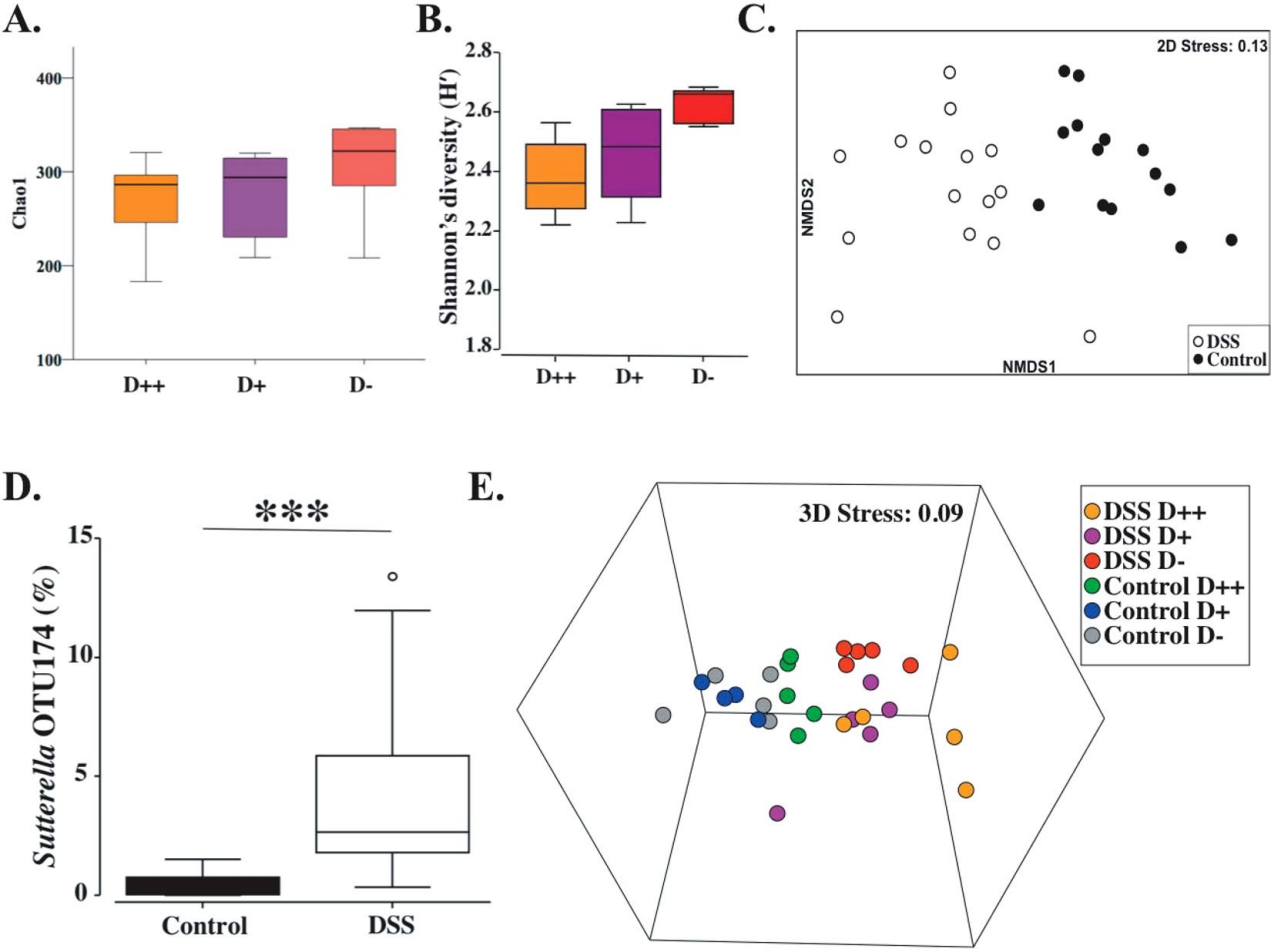
Microbial composition of faecal samples at Day 7 from DSS-treated mice. (**A**) Species richness (measured by chao1), n = 5/group. (**B**) Shannon’s diversity (H′), n = 5/group. (**C**) Non-metric multidimensional scaling (NMDS) plot of the Bray-Curtis resemblance matrix following square-root transformation of relative abundance data showing the impact of DSS on the overall microbial composition, confirmed by pair-wise PERMANOVA (Control vs DSS): t = 3.34, p = 0.01, permutations = 999, n = 15/group (with and without nesting for vitamin D subgrouping). (**D**) Relative abundance (%) of *Sutterella* OTU174 between control and DSS mice (LDA 4.27, P < 0.0001), p-value derived from Linear discriminant analysis effect size (LEfSe), n = 15/group. (**E**) NMDS plot of the Bray-Curtis resemblance matrix following square-root transformation of relative abundance data demonstrating a significant shift of the control D++ microbial composition (green) towards that of the DSS group, n = 5/group. ^***^P < *0.001*.

Treatment with DSS reduced the number of operational taxonomic units (OTUs) within samples analysed at day 7 from the D− (*P* = 0.09) and D+ (*P* = 0.04) but not D++ group relative to D− controls (not shown), but there was no significant difference between the DSS groups (Fig. 7A). DSS did not affect other measures of α-diversity, in particular species evenness and Shannon’s diversity (Fig. 7B, Supplementary Fig. 3D). However, DSS had a significant impact on overall microbial composition (β-diversity) at day 7 (Fig. 7C). Further, 111 microbial taxa at all taxonomic levels were found to be differentially abundant between controls and DSS mice using LEfSe analysis (Supplementary Table Ib). There was enrichment with DSS of disease-associated Proteobacteria and a reduction in taxa belonging to Firmicutes.

Notably, *Sutterella* OTU174 increased in relative abundance in DSS mice as compared to controls (LDA score: 4.27, p < 0.0001) (Fig. 7D, Supplementary Table Ia). This is relevant given similar rise of *Sutterella* in non-DSS treated controls from the D++ group, Fig. 6C, suggesting a shift in faecal microbiome of D++ controls to that of DSS mice. Further, when examining the overall microbiome composition there is a clear shift for D++ mice towards that of DSS mice (Fig. 7E).

## Discussion

This is the first study to examine the effect of high dose vitamin D supplementation in an IBD model. High dose vitamin D supplementation led to more severe DSS colitis as measured by blinded endoscopic and histologic assessment, weight loss and fall in serum albumin.

The development of colitis was associated with an acute drop in serum 25(OH)D and 1,25(OH)_2_D levels by a mean of 62% among the high vitamin D group and 23% among the vitamin D sufficient group. Other groups have demonstrated a drop in 1,25(OH)_2_D_3_ with DSS colitis but not in 25(OH)D_3_^5^. This is most likely because the change in 25(OH)D_3_ was most apparent in the high vitamin D group where the baseline 25(OH)D_3_ level was significantly greater, this diet has not been examined in the prior studies of DSS colitis. The drop in 25(OH)D_3_ was associated with a greater than five-fold increase in gene expression of kidney CYP24A1 among D+ mice, an enzyme responsible for the catabolism of both 25 and 1,25(OH)_2_D. This suggests that vitamin D metabolites drops in response to inflammation. We did not observe this increase in kidney CYP24A1 expression in the D++ group which had the greatest drop in 25(OH)D, likely due to increased expression at baseline as a counter-regulatory mechanism to the higher 25(OH)D levels. An alternative mechanism must exist to explain the fall in vitamin D levels in this group. It is well established that 1,25(OH)_2_D can be produced by colonic epithelial cells^30^ and monocytes^31^, and this is also regulated by local CYP27B1 and CYP24A1. In fact, a study by Liu *et al*., demonstrated increased Cyp24A1 expression from the proximal, but not distal, colon with DSS colitis^15^. Thus, it is possible that with the colon inflammation, colonic epithelial cell and monocyte CYP24A1 expression is upregulated leading to the fall in serum 25(OH)D levels. The recovery of 25(OH)D_3_ was slow and did not return to baseline by day 35 in the D++ group despite resolution of colitis. This is likely due to this group having the greatest drop in 25(OH)D_3_, thus the time taken for 25(OH)D_3_ to return to baseline would be expected to take the longest even after the colitis has resolved.

In critical illness, reduced 25(OH)D and 1,25(OH)_2_D levels have been observed, however this has been in association with reduced concentrations of the VDBP^32^. In our model we observed an increase in VDBP levels. While this could simply be a discrepancy between mouse and human vitamin D metabolism, it may also be unique to intestinal inflammation. In our previous work examining over 300 patients with CD in remission, high VDBP concentrations were independently associated with a 20% increased risk of subsequent clinical relapse of disease^33^. Thus, subclinical inflammation may lead to increased VDBP levels and subsequent disease flare. VDBP has an important role in actin scavenging such that in the case of acute tissue damage, cellular actin will bind plasma VDBP for subsequent rapid clearance of the complex^34^. It is possible that in response to intestinal tissue damage, VDBP was upregulated and thus is a biomarker of subclinical disease activity.

Contrary to previous animal studies, vitamin D deficiency did not predispose mice to worse colitis, though the measurement of colitis severity by endoscopy in this study differed to the clinical measurements reported in other studies. We did however observe transient weight loss and a trend for increased histological inflammation of the colon in the vitamin D-deficient group consistent with other reports. Previously reported rodents with increased susceptibility to DSS colitis have included vitamin D receptor- and Cyp27B1- KO mice. However, they had absolute vitamin D deficiency and numerous complicating skeletal abnormalities such that they may not be a good representation of the human condition^4,5^. Animal models of dietary-induced vitamin D deficiency better mimic clinical deficiency observed in humans; however, mixed results have been reported in these vitamin D deficient mice^3,35^.

Serum cytokines, TNF-α, IFN-γ, IL-10, IL-6, IL-12p40 and IL-1β, increased with the induction of DSS colitis, and is consistent with previous work from our group^16^. No differences were seen in serum cytokine levels between the different vitamin D groups, with the exception of IL-12p40 which was higher among colitic mice from the D++ group compared to the vitamin D-deficient group. It is possible that other cytokine levels reached a plateau level in all DSS groups, making it impossible to detect differences between the different vitamin D dietary groups. We have previously reviewed the effect of vitamin D supplementation *in-vivo* and *in-vitro* on innate and adaptive immune cells responses, particularly with respect to gastrointestinal inflammation^36^. In summary, vitamin D supplementation can have favourable effects in preserving intestinal epithelial barrier function, production of anti-microbial peptides, attenuating capacity for antigen presentation, reducing dendritic cell maturation and T-cell proliferation. The effect of different doses of vitamin D on these responses was not examined in this study, but it would be important in future work to identify those responses that reversed with high vitamin D supplementation.

The mechanism by which high dose vitamin D supplementation increased colitis susceptibility remains unclear, but we did observe that vitamin D had a potent effect on the microbial composition of faeces from plain-water treated, control mice. Interestingly, the microbial composition of faeces from D++ control mice approached that of DSS mice, suggesting a shift to a more pro-inflammatory microbiome even before starting DSS treatment. Some of the changes seen across vitamin D categories were confirmed by correlation with serum 25(OH)D_3_ levels. Most notably there was a consistent increase in *Sutterella* spp. This same organism was also enriched in mice with colitis. *Sutterella* spp. have been reported to be enriched in human subjects with inflammatory bowel diseases^37^ and a recent study of faecal microbiota transplantation in ulcerative colitis found recipients with increases in *Sutterella* spp. were consistently less likely to respond to the treatment^38^. Similarly, the group Bacteroidales S24-7 showed a strong positive correlation with 25(OH)D_3_ levels but was depleted in the DSS mice. It is possible that the significant bloom in this taxon in D++ mice impacts microbiome stability and, in turn, susceptibility to the effects of DSS, thus potentially predisposing these mice to more severe colitis.

It remains unclear if the observed changes in faecal microbiota are a direct response to changes in vitamin D, or mediated indirectly through changes in mucosal immune responses. While our findings demonstrate an association between vitamin D dosing, faecal microbiota changes and susceptibility to colitis, we acknowledge that a specifically designed study would be needed to draw definitive conclusions about cause and effect.

In conclusion, high dose vitamin D worsens the severity of murine colitis induced by DSS, and is associated with distinct changes in microbial composition that may be a direct dietary effect or as a result of dysregulation of the gut mucosal immune response. Future work needs to further explore the effects of high levels of vitamin D on gut mucosal immunity to better understand if high as well as low vitamin D levels lead to a dysregulation.

## Electronic supplementary material

Supplementary figures
