# Supplementary material for: High Dose Vitamin D supplementation alters faecal microbiome and predisposes mice to more severe colitis
Source: Sci Rep. 2018 Jul 31;8:11511. doi: 10.1038/s41598-018-29759-y (PMC6068189; doi:10.1038/s41598-018-29759-y)
Supplement: Supplementary file 1 — Supplementary figures [file 41598_2018_29759_MOESM1_ESM.docx]

**SUPPLEMENTARY FILE**

**High Dose Vitamin D supplementation alters faecal microbiome and predisposes mice to more severe colitis**

**Simon Ghaly**^1,2,3^**^*^,** Nadeem O Kaakoush^4^, Frances Lloyd^2^, Terence McGonigle^1^, Danny Mok^1^, Angela Baird^1^, Borut Klopcic^1^, Lavinia Gordon^5^, Shelley Gorman^1^, Cynthia Forest^6^, Roger Bouillon^7^, Ian C Lawrance^2,8^, Prue H Hart^1^

^1^Telethon Kids Institute, The University of Western Australia, Perth, WA, Australia

**^2^** School of Medicine and Pharmacology, The University of Western Australia, Perth, WA, Australia

**^3^**Department of Gastroenterology and Hepatology, St. Vincent’s Hospital, Sydney, NSW, Australia

^4^School of Medical Sciences, UNSW Sydney, Kensington, NSW, Australia

^5^Australian Genome Research Facility, The Walter and Eliza Hall Institute, Parkville, Victoria, Australia

^6^ Department of Anatomical Pathology, PathWest, Fiona Stanley Hospital, Murdoch, WA, Australia

**^7^**Clinical and Experimental Endocrinology, Katholieke Universiteit Leuven, Leuven, Belgium

^8^Centre for Inflammatory Bowel Disease, St. John of God Hospital, Subiaco, WA, Australia


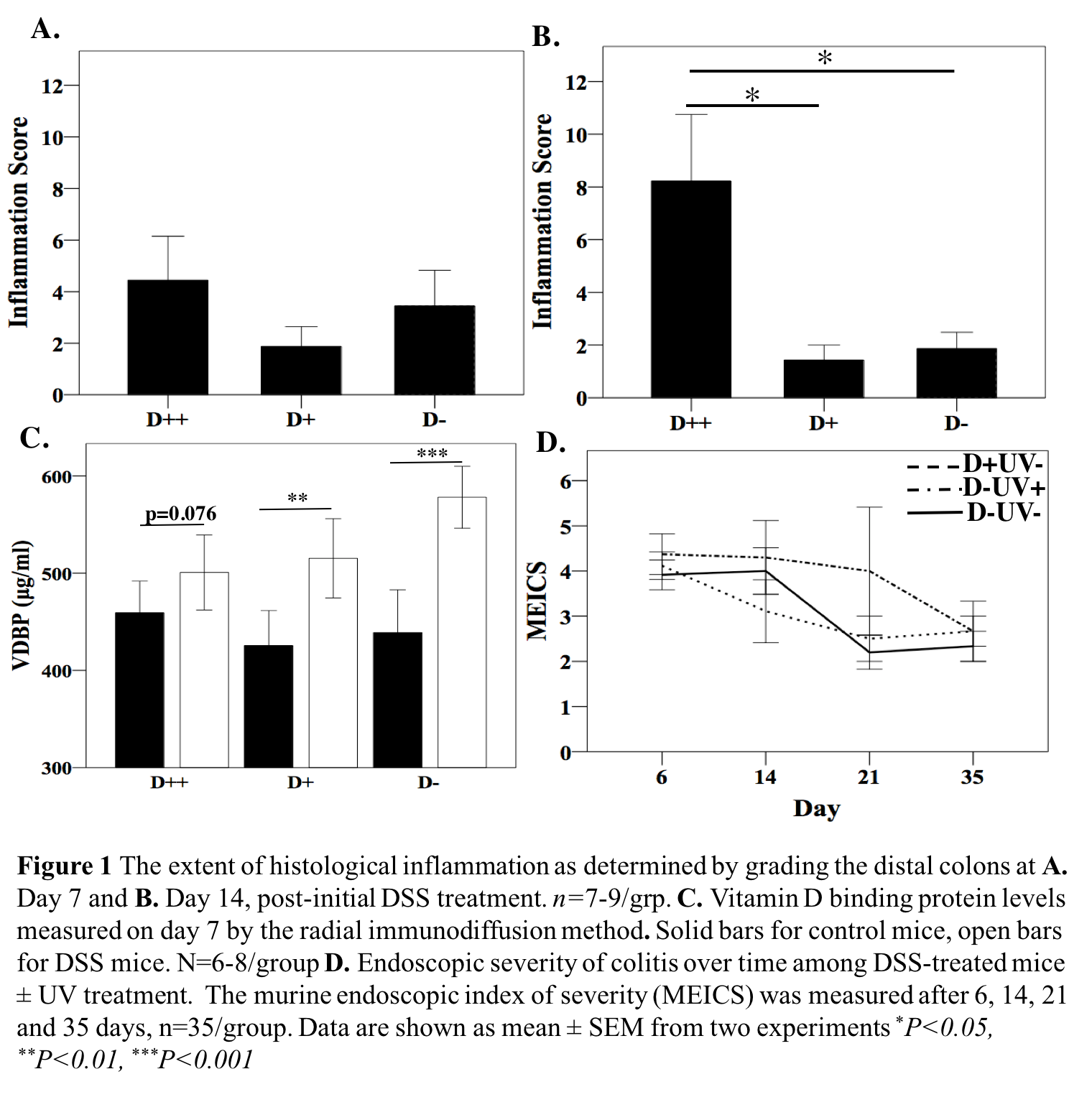


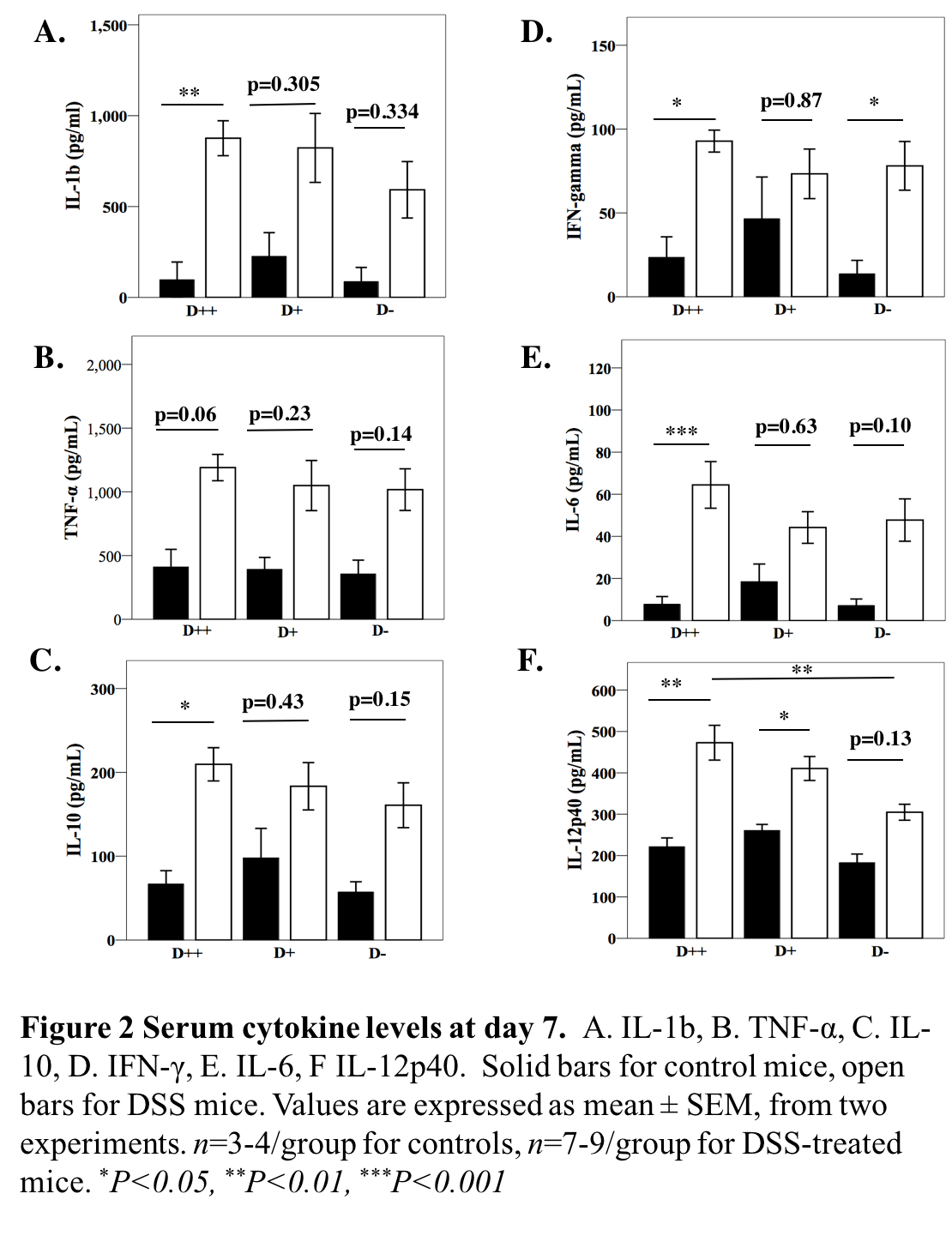


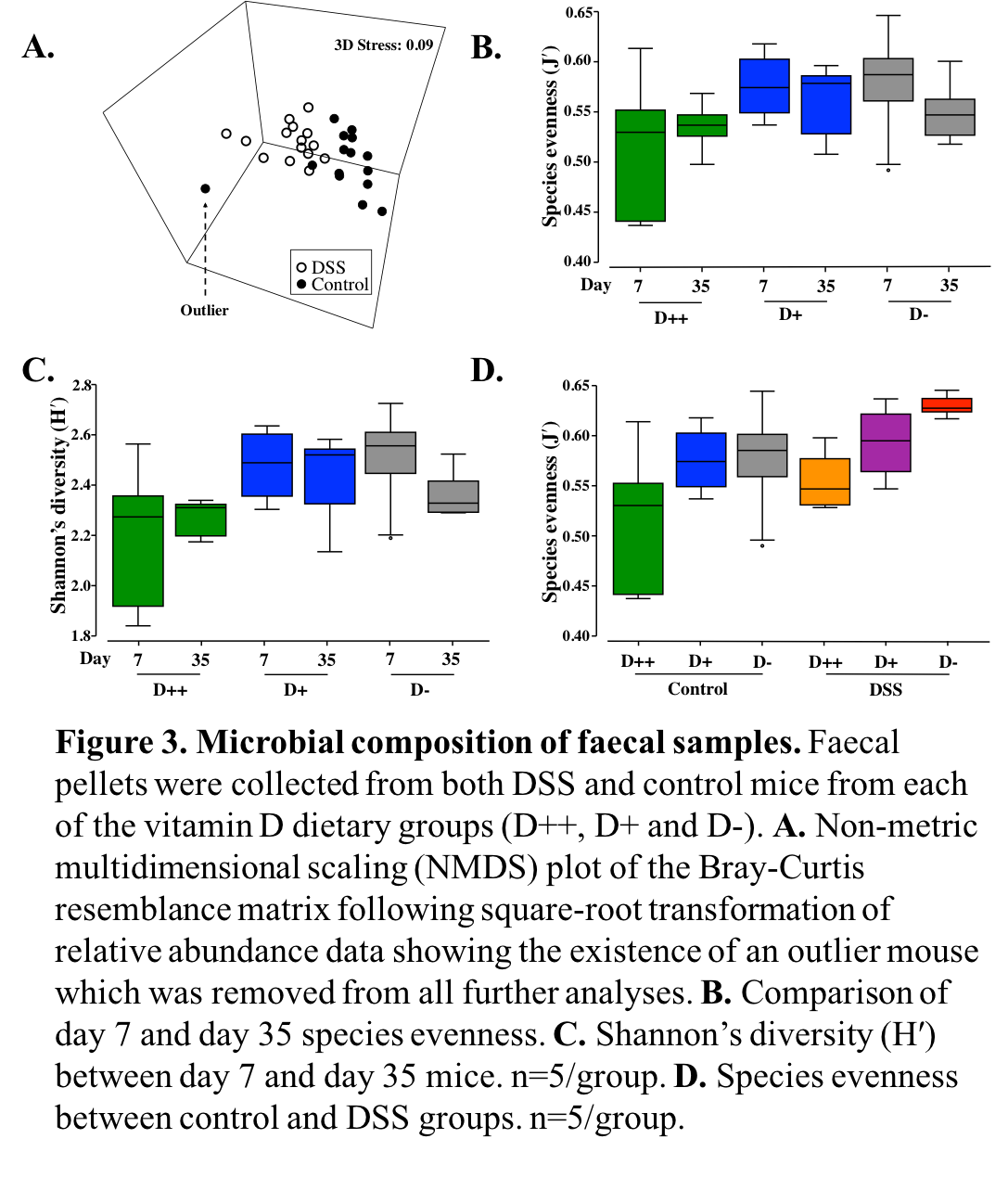


| **Microbial taxa** | **Group** | **LDA Score** | **P-value** |
| --- | --- | --- | --- |
| Bacteria.Actinobacteria.Actinobacteriia.Actinomycetales.Actinomycetales_unclassified.Actinomycetales_unclassified.OTU18 | D+ | 3.96 | 0.047 |
| Bacteria.Actinobacteria.Coriobacteriia | D- | 3.33 | 0.016 |
| Bacteria.Actinobacteria.Coriobacteriia.Coriobacteriales | D- | 3.33 | 0.016 |
| Bacteria.Actinobacteria.Coriobacteriia.Coriobacteriales.Coriobacteriaceae | D- | 3.33 | 0.016 |
| Bacteria.Actinobacteria.Coriobacteriia.Coriobacteriales.Coriobacteriaceae.Coriobacteriaceae_unclassified | D- | 3.30 | 0.011 |
| Bacteria.Actinobacteria.Coriobacteriia.Coriobacteriales.Coriobacteriaceae.Coriobacteriaceae_unclassified.OTU30 | D- | 3.30 | 0.011 |
| Bacteria.Bacteroidetes | D++ | 5.16 | 0.011 |
| Bacteria.Bacteroidetes.Bacteroidia | D++ | 5.17 | 0.011 |
| Bacteria.Bacteroidetes.Bacteroidia.Bacteroidales | D++ | 5.16 | 0.011 |
| Bacteria.Bacteroidetes.Bacteroidia.Bacteroidales.Rikenellaceae | D++ | 3.96 | 0.020 |
| Bacteria.Bacteroidetes.Bacteroidia.Bacteroidales.Rikenellaceae.PW3 | D++ | 4.10 | 0.008 |
| Bacteria.Bacteroidetes.Bacteroidia.Bacteroidales.Rikenellaceae.PW3.OTU57 | D++ | 4.10 | 0.008 |
| Bacteria.Bacteroidetes.Bacteroidia.Bacteroidales.Rikenellaceae.Rikenellaceae_unclassified | D++ | 3.97 | 0.020 |
| Bacteria.Bacteroidetes.Bacteroidia.Bacteroidales.Rikenellaceae.Rikenellaceae_unclassified.OTU55 | D++ | 3.95 | 0.020 |
| Bacteria.Bacteroidetes.Flavobacteriia | D++ | 4.16 | 0.014 |
| Bacteria.Bacteroidetes.Flavobacteriia.Flavobacteriales | D++ | 4.17 | 0.014 |
| Bacteria.Bacteroidetes.Flavobacteriia.Flavobacteriales.Flavobacteriales_unclassified | D++ | 4.22 | 0.014 |
| Bacteria.Bacteroidetes.Flavobacteriia.Flavobacteriales.Flavobacteriales_unclassified.Flavobacteriales_unclassified | D++ | 4.20 | 0.014 |
| Bacteria.Bacteroidetes.Flavobacteriia.Flavobacteriales.Flavobacteriales_unclassified.Flavobacteriales_unclassified.OTU64 | D++ | 4.21 | 0.020 |
| Bacteria.Firmicutes | D+ | 5.21 | 0.009 |
| Bacteria.Firmicutes.Clostridia | D+ | 5.14 | 0.010 |
| Bacteria.Firmicutes.Clostridia.Clostridiales | D+ | 5.13 | 0.010 |
| Bacteria.Firmicutes.Clostridia.Clostridiales.Clostridiales_unclassified | D+ | 4.95 | 0.020 |
| Bacteria.Firmicutes.Clostridia.Clostridiales.Clostridiales_unclassified.Clostridiales_unclassified | D+ | 4.94 | 0.020 |
| Bacteria.Firmicutes.Clostridia.Clostridiales.Clostridiales_unclassified.Clostridiales_unclassified.OTU105 | D+ | 4.88 | 0.021 |
| Bacteria.Firmicutes.Clostridia.Clostridiales.Dehalobacteriaceae | D+ | 2.92 | 0.031 |
| Bacteria.Firmicutes.Clostridia.Clostridiales.Dehalobacteriaceae.Dehalobacterium | D+ | 2.94 | 0.031 |
| Bacteria.Firmicutes.Clostridia.Clostridiales.Dehalobacteriaceae.Dehalobacterium.OTU114 | D+ | 2.93 | 0.031 |
| Bacteria.Firmicutes.Clostridia.Clostridiales.Lachnospiraceae | D- | 4.47 | 0.010 |
| Bacteria.Firmicutes.Clostridia.Clostridiales.Lachnospiraceae.Coprococcus | D- | 4.01 | 0.024 |
| Bacteria.Firmicutes.Clostridia.Clostridiales.Lachnospiraceae.Coprococcus.OTU118 | D- | 4.01 | 0.024 |
| Bacteria.Firmicutes.Clostridia.Clostridiales.Lachnospiraceae.Lachnospiraceae_unclassified | D- | 4.29 | 0.023 |
| Bacteria.Firmicutes.Clostridia.Clostridiales.Lachnospiraceae.Lachnospiraceae_unclassified.OTU115 | D- | 4.19 | 0.024 |
| Bacteria.Firmicutes.Clostridia.Clostridiales.Lachnospiraceae.Lachnospiraceae_unclassified.OTU116 | D- | 3.61 | 0.034 |
| Bacteria.Firmicutes.Clostridia.Clostridiales.Ruminococcaceae | D+ | 4.48 | 0.030 |
| Bacteria.Firmicutes.Clostridia.Clostridiales.Ruminococcaceae.Oscillospira | D+ | 4.37 | 0.024 |
| Bacteria.Firmicutes.Clostridia.Clostridiales.Ruminococcaceae.Oscillospira.OTU127 | D+ | 4.41 | 0.024 |
| Bacteria.Proteobacteria.Alphaproteobacteria | D++ | 3.49 | 0.048 |
| Bacteria.TM7.TM7_3.TM7_3_unclassified.TM7_3_unclassified.TM7_3_unclassified.OTU230 | D+ | 3.89 | 0.044 |
| Bacteria.TM7.TM7_3.TM7_3_unclassified.TM7_3_unclassified.TM7_3_unclassified.OTU231 | D+ | 4.09 | 0.047 |

**Table Ia LEfSe analyses comparing control mice from different vitamin D groups.**

Threshold used was linear discriminant analysis (LDA) score >2 and P<0.05. The results provide a list of bacterial taxa at all taxonomic levels that are differentially abundant between the three vitamin D groups in control mice.

| **Microbial taxa** | **Group** | **LDA Score** | **P-value** |
| --- | --- | --- | --- |
| Bacteria.Actinobacteria.Actinobacteria_unclassified | DSS | 2.65 | 0.003 |
| Bacteria.Actinobacteria.Actinobacteria_unclassified.Actinobacteria_unclassified | DSS | 2.65 | 0.003 |
| Bacteria.Actinobacteria.Actinobacteria_unclassified.Actinobacteria_unclassified.Actinobacteria_unclassified | DSS | 2.65 | 0.003 |
| Bacteria.Actinobacteria.Actinobacteria_unclassified.Actinobacteria_unclassified.Actinobacteria_unclassified.Actinobacteria_unclassified | DSS | 2.65 | 0.003 |
| Bacteria.Actinobacteria.Actinobacteria_unclassified.Actinobacteria_unclassified.Actinobacteria_unclassified.Actinobacteria_unclassified.OTU14 | DSS | 2.65 | 0.003 |
| Bacteria.Actinobacteria.Actinobacteriia.Actinomycetales.Actinomycetales_unclassified | Control | 3.27 | 0.011 |
| Bacteria.Actinobacteria.Actinobacteriia.Actinomycetales.Actinomycetales_unclassified.Actinomycetales_unclassified | Control | 3.27 | 0.011 |
| Bacteria.Actinobacteria.Actinobacteriia.Actinomycetales.Actinomycetales_unclassified.Actinomycetales_unclassified.OTU17 | Control | 3.22 | 0.003 |
| Bacteria.Actinobacteria.Coriobacteriia.Coriobacteriales.Coriobacteriaceae.Adlercreutzia | Control | 2.61 | 0.021 |
| Bacteria.Actinobacteria.Coriobacteriia.Coriobacteriales.Coriobacteriaceae.Adlercreutzia.OTU31 | Control | 2.61 | 0.021 |
| Bacteria.Bacteroidetes.Bacteroidia.Bacteroidales._Paraprevotellaceae_ | Control | 3.36 | <0.001 |
| Bacteria.Bacteroidetes.Bacteroidia.Bacteroidales._Paraprevotellaceae_.YRC22 | Control | 3.36 | <0.001 |
| Bacteria.Bacteroidetes.Bacteroidia.Bacteroidales._Paraprevotellaceae_.YRC22.OTU62 | Control | 3.36 | <0.001 |
| Bacteria.Bacteroidetes.Bacteroidia.Bacteroidales.Bacteroidaceae | DSS | 4.78 | 0.002 |
| Bacteria.Bacteroidetes.Bacteroidia.Bacteroidales.Bacteroidaceae.Bacteroides | DSS | 4.78 | 0.002 |
| Bacteria.Bacteroidetes.Bacteroidia.Bacteroidales.Bacteroidales_unclassified.Bacteroidales_unclassified.OTU35 | DSS | 4.12 | <0.001 |
| Bacteria.Bacteroidetes.Bacteroidia.Bacteroidales.Porphyromonadaceae | DSS | 4.65 | 0.002 |
| Bacteria.Bacteroidetes.Bacteroidia.Bacteroidales.Porphyromonadaceae.Parabacteroides | DSS | 4.61 | 0.003 |
| Bacteria.Bacteroidetes.Bacteroidia.Bacteroidales.Porphyromonadaceae.Parabacteroides.OTU48 | DSS | 4.00 | 0.002 |
| Bacteria.Bacteroidetes.Bacteroidia.Bacteroidales.Porphyromonadaceae.Parabacteroides.OTU49 | DSS | 4.49 | 0.013 |
| Bacteria.Bacteroidetes.Bacteroidia.Bacteroidales.Porphyromonadaceae.Porphyromonadaceae_unclassified | DSS | 2.99 | 0.001 |
| Bacteria.Bacteroidetes.Bacteroidia.Bacteroidales.Porphyromonadaceae.Porphyromonadaceae_unclassified.OTU43 | DSS | 2.99 | 0.001 |
| Bacteria.Bacteroidetes.Bacteroidia.Bacteroidales.Rikenellaceae | Control | 3.78 | 0.023 |
| Bacteria.Bacteroidetes.Bacteroidia.Bacteroidales.Rikenellaceae.Rikenellaceae_unclassified | Control | 3.78 | 0.023 |
| Bacteria.Bacteroidetes.Bacteroidia.Bacteroidales.Rikenellaceae.Rikenellaceae_unclassified.OTU55 | Control | 3.79 | 0.023 |
| Bacteria.Bacteroidetes.Bacteroidia.Bacteroidales.S24_7 | Control | 4.94 | <0.001 |
| Bacteria.Bacteroidetes.Bacteroidia.Bacteroidales.S24_7.S24_7_unclassified | Control | 4.94 | <0.001 |
| Bacteria.Bacteroidetes.Bacteroidia.Bacteroidales.S24_7.S24_7_unclassified.OTU58 | Control | 4.94 | <0.001 |
| Bacteria.Deferribacteres | DSS | 3.24 | 0.001 |
| Bacteria.Deferribacteres.Deferribacteres | DSS | 3.24 | 0.001 |
| Bacteria.Deferribacteres.Deferribacteres.Deferribacterales | DSS | 3.24 | 0.001 |
| Bacteria.Deferribacteres.Deferribacteres.Deferribacterales.Deferribacteraceae | DSS | 3.24 | 0.001 |
| Bacteria.Deferribacteres.Deferribacteres.Deferribacterales.Deferribacteraceae.Mucispirillum | DSS | 3.24 | 0.001 |
| Bacteria.Deferribacteres.Deferribacteres.Deferribacterales.Deferribacteraceae.Mucispirillum.OTU81 | DSS | 3.24 | 0.001 |
| Bacteria.Firmicutes | Control | 4.96 | 0.001 |
| Bacteria.Firmicutes.Bacilli | Control | 4.10 | <0.001 |
| Bacteria.Firmicutes.Bacilli.Bacillales | Control | 3.01 | 0.012 |
| Bacteria.Firmicutes.Bacilli.Lactobacillales | Control | 4.11 | <0.001 |
| Bacteria.Firmicutes.Bacilli.Lactobacillales.Enterococcaceae | Control | 2.75 | 0.015 |
| Bacteria.Firmicutes.Bacilli.Lactobacillales.Enterococcaceae.Enterococcus | Control | 2.75 | 0.015 |
| Bacteria.Firmicutes.Bacilli.Lactobacillales.Enterococcaceae.Enterococcus.OTU95 | Control | 2.75 | 0.015 |
| Bacteria.Firmicutes.Bacilli.Lactobacillales.Lactobacillaceae | Control | 4.08 | 0.001 |
| Bacteria.Firmicutes.Bacilli.Lactobacillales.Lactobacillaceae.Lactobacillus | Control | 4.08 | 0.001 |
| Bacteria.Firmicutes.Bacilli.Lactobacillales.Lactobacillaceae.Lactobacillus.OTU97 | Control | 4.07 | <0.001 |
| Bacteria.Firmicutes.Bacilli.Lactobacillales.Streptococcaceae | Control | 2.56 | 0.001 |
| Bacteria.Firmicutes.Bacilli.Lactobacillales.Streptococcaceae.Streptococcus | Control | 2.56 | 0.001 |
| Bacteria.Firmicutes.Bacilli.Lactobacillales.Streptococcaceae.Streptococcus.OTU100 | Control | 2.56 | 0.001 |
| Bacteria.Firmicutes.Clostridia | Control | 4.95 | 0.001 |
| Bacteria.Firmicutes.Clostridia.Clostridiales | Control | 4.95 | 0.001 |
| Bacteria.Firmicutes.Clostridia.Clostridiales.Clostridiaceae | DSS | 3.38 | 0.016 |
| Bacteria.Firmicutes.Clostridia.Clostridiales.Clostridiales_unclassified | Control | 4.75 | <0.001 |
| Bacteria.Firmicutes.Clostridia.Clostridiales.Clostridiales_unclassified.Clostridiales_unclassified | Control | 4.75 | <0.001 |
| Bacteria.Firmicutes.Clostridia.Clostridiales.Clostridiales_unclassified.Clostridiales_unclassified.OTU105 | Control | 4.75 | <0.001 |
| Bacteria.Firmicutes.Clostridia.Clostridiales.Dehalobacteriaceae | Control | 2.58 | 0.009 |
| Bacteria.Firmicutes.Clostridia.Clostridiales.Dehalobacteriaceae.Dehalobacterium | Control | 2.58 | 0.009 |
| Bacteria.Firmicutes.Clostridia.Clostridiales.Dehalobacteriaceae.Dehalobacterium.OTU114 | Control | 2.58 | 0.009 |
| Bacteria.Firmicutes.Clostridia.Clostridiales.Lachnospiraceae | Control | 4.18 | 0.008 |
| Bacteria.Firmicutes.Clostridia.Clostridiales.Lachnospiraceae.Butyrivibrio | Control | 2.74 | <0.001 |
| Bacteria.Firmicutes.Clostridia.Clostridiales.Lachnospiraceae.Butyrivibrio.OTU117 | Control | 2.75 | <0.001 |
| Bacteria.Firmicutes.Clostridia.Clostridiales.Lachnospiraceae.Lachnospiraceae_unclassified | Control | 4.13 | <0.001 |
| Bacteria.Firmicutes.Clostridia.Clostridiales.Lachnospiraceae.Lachnospiraceae_unclassified.OTU115 | Control | 3.98 | 0.002 |
| Bacteria.Firmicutes.Clostridia.Clostridiales.Lachnospiraceae.Lachnospiraceae_unclassified.OTU116 | Control | 3.60 | <0.001 |
| Bacteria.Firmicutes.Clostridia.Clostridiales.Ruminococcaceae | Control | 4.23 | 0.004 |
| Bacteria.Firmicutes.Clostridia.Clostridiales.Ruminococcaceae.Oscillospira | Control | 4.16 | 0.002 |
| Bacteria.Firmicutes.Clostridia.Clostridiales.Ruminococcaceae.Oscillospira.OTU127 | Control | 4.16 | 0.002 |
| Bacteria.Firmicutes.Clostridia.Clostridiales.Ruminococcaceae.Ruminococcaceae_unclassified.OTU125 | Control | 2.79 | 0.005 |
| Bacteria.Firmicutes.Clostridia.Clostridiales.Ruminococcaceae.Ruminococcus | Control | 3.06 | 0.011 |
| Bacteria.Firmicutes.Clostridia.Clostridiales.Ruminococcaceae.Ruminococcus.OTU129 | Control | 3.06 | 0.011 |
| Bacteria.Firmicutes.Erysipelotrichi | DSS | 3.99 | 0.008 |
| Bacteria.Firmicutes.Erysipelotrichi.Erysipelotrichales | DSS | 3.99 | 0.008 |
| Bacteria.Firmicutes.Erysipelotrichi.Erysipelotrichales.Erysipelotrichaceae | DSS | 3.99 | 0.008 |
| Bacteria.Firmicutes.Erysipelotrichi.Erysipelotrichales.Erysipelotrichaceae.Erysipelotrichaceae_unclassified | DSS | 3.96 | 0.001 |
| Bacteria.Firmicutes.Erysipelotrichi.Erysipelotrichales.Erysipelotrichaceae.Erysipelotrichaceae_unclassified.OTU147 | DSS | 3.98 | <0.001 |
| Bacteria.Proteobacteria | DSS | 4.67 | <0.001 |
| Bacteria.Proteobacteria.Alphaproteobacteria.Alphaproteobacteria_unclassified | Control | 2.62 | 0.002 |
| Bacteria.Proteobacteria.Alphaproteobacteria.Alphaproteobacteria_unclassified.Alphaproteobacteria_unclassified | Control | 2.62 | 0.002 |
| Bacteria.Proteobacteria.Alphaproteobacteria.Alphaproteobacteria_unclassified.Alphaproteobacteria_unclassified.Alphaproteobacteria_unclassified | Control | 2.62 | 0.002 |
| Bacteria.Proteobacteria.Alphaproteobacteria.Alphaproteobacteria_unclassified.Alphaproteobacteria_unclassified.Alphaproteobacteria_unclassified.OTU159 | Control | 2.62 | 0.002 |
| Bacteria.Proteobacteria.Betaproteobacteria | DSS | 4.22 | <0.001 |
| Bacteria.Proteobacteria.Betaproteobacteria.Burkholderiales | DSS | 4.22 | <0.001 |
| Bacteria.Proteobacteria.Betaproteobacteria.Burkholderiales.Alcaligenaceae | DSS | 4.22 | <0.001 |
| Bacteria.Proteobacteria.Betaproteobacteria.Burkholderiales.Alcaligenaceae.Sutterella | DSS | 4.22 | <0.001 |
| Bacteria.Proteobacteria.Betaproteobacteria.Burkholderiales.Alcaligenaceae.Sutterella.OTU174 | DSS | 4.22 | <0.001 |
| Bacteria.Proteobacteria.Deltaproteobacteria.Desulfovibrionales.Desulfovibrionaceae.Desulfovibrionaceae_unclassified | Control | 3.02 | <0.001 |
| Bacteria.Proteobacteria.Deltaproteobacteria.Desulfovibrionales.Desulfovibrionaceae.Desulfovibrionaceae_unclassified.OTU186 | Control | 3.02 | <0.001 |
| Bacteria.Proteobacteria.Epsilonproteobacteria | DSS | 3.11 | <0.001 |
| Bacteria.Proteobacteria.Epsilonproteobacteria.Campylobacterales | DSS | 3.11 | <0.001 |
| Bacteria.Proteobacteria.Epsilonproteobacteria.Campylobacterales.Helicobacteraceae | DSS | 3.11 | <0.001 |
| Bacteria.Proteobacteria.Epsilonproteobacteria.Campylobacterales.Helicobacteraceae.Flexispira | DSS | 3.10 | <0.001 |
| Bacteria.Proteobacteria.Epsilonproteobacteria.Campylobacterales.Helicobacteraceae.Flexispira.OTU194 | DSS | 3.10 | <0.001 |
| Bacteria.Proteobacteria.Gammaproteobacteria | DSS | 4.39 | 0.001 |
| Bacteria.Proteobacteria.Gammaproteobacteria.Enterobacteriales | DSS | 4.39 | 0.001 |
| Bacteria.Proteobacteria.Gammaproteobacteria.Enterobacteriales.Enterobacteriaceae | DSS | 4.39 | 0.001 |
| Bacteria.Proteobacteria.Gammaproteobacteria.Enterobacteriales.Enterobacteriaceae.Enterobacteriaceae_unclassified | DSS | 4.39 | 0.001 |
| Bacteria.Proteobacteria.Gammaproteobacteria.Enterobacteriales.Enterobacteriaceae.Enterobacteriaceae_unclassified.OTU203 | DSS | 4.39 | 0.001 |
| Bacteria.Proteobacteria.Gammaproteobacteria.Xanthomonadales | Control | 2.94 | <0.001 |
| Bacteria.Proteobacteria.Gammaproteobacteria.Xanthomonadales.Sinobacteraceae | Control | 2.86 | 0.010 |
| Bacteria.Proteobacteria.Gammaproteobacteria.Xanthomonadales.Sinobacteraceae.Sinobacteraceae_unclassified | Control | 2.92 | 0.010 |
| Bacteria.Proteobacteria.Gammaproteobacteria.Xanthomonadales.Sinobacteraceae.Sinobacteraceae_unclassified.OTU219 | Control | 2.89 | 0.010 |
| Bacteria.Tenericutes | Control | 2.69 | <0.001 |
| Bacteria.Tenericutes.Mollicutes | Control | 2.69 | <0.001 |
| Bacteria.Tenericutes.Mollicutes.RF39 | Control | 2.69 | <0.001 |
| Bacteria.Tenericutes.Mollicutes.RF39.RF39_unclassified | Control | 2.69 | <0.001 |
| Bacteria.Tenericutes.Mollicutes.RF39.RF39_unclassified.RF39_unclassified | Control | 2.69 | <0.001 |
| Bacteria.Tenericutes.Mollicutes.RF39.RF39_unclassified.RF39_unclassified.OTU234 | Control | 2.69 | <0.001 |
| Bacteria.TM7 | Control | 3.50 | 0.007 |
| Bacteria.TM7.TM7_3 | Control | 3.50 | 0.006 |
| Bacteria.TM7.TM7_3.CW040 | Control | 3.50 | 0.005 |
| Bacteria.TM7.TM7_3.CW040.F16 | Control | 3.50 | 0.005 |
| Bacteria.TM7.TM7_3.CW040.F16.F16_unclassified | Control | 3.50 | 0.005 |
| Bacteria.TM7.TM7_3.CW040.F16.F16_unclassified.OTU232 | Control | 3.50 | 0.005 |

**Table Ib. LEfSe analyses comparing DSS to control mice.**

Dietary vitamin D grouping was nested as a subclass. Threshold used was linear discriminant analysis (LDA) score >2 and P<0.05. The results provide a list of bacterial taxa at all taxonomic levels that are differentially abundant between DSS and control mice.
